# Supplementary material for: Two energy barriers and a transient intermediate state determine the unfolding and folding dynamics of cold shock protein
Source: Commun Chem. 2021 Nov 9;4:156. doi: 10.1038/s42004-021-00592-1 (PMC9814876; doi:10.1038/s42004-021-00592-1)
Supplement: Supplementary file 1 — Supplemental Material [file 42004_2021_592_MOESM1_ESM.pdf]

Supplementary Information

“Two energy barriers and a transient intermediate state determine the unfolding and folding dynamics of cold shock protein”

Haiyan Hong[1#], Zilong Guo[1,2,3#], Hao Sun[1], Ping Yu[1], Huanhuan Su[1], Xuening Ma[1], Hu Chen[1,2,3*]

[1] Research Institute for Biomimetics and Soft Matter, Fujian Provincial Key Lab for Soft Functional Materials Research, Department of Physics, Xiamen University, Xiamen 361005, China

[2]Center of Biomedical Physics, Wenzhou Institute, University of Chinese Academy of Sciences, Wenzhou, China 325000

[3]Oujiang Laboratory, Wenzhou, Zhejiang 325000, China

# Contributed equally to this work

E-mail: chenhu@xmu.edu.cn

Supplementary Note 1

NIU Model and Fitting Parameters

As shown in Fig. 4a and 4b in main text, logarithm of unfolding rate at different force range gives different slopes. We fit force-dependent unfolding rate over full force range according to the model proposed by Olga K. Dudko[1]. Unfolding rate of Csp k_u, can be written as:

$$k_u = \frac{k_{\mathrm{IU}} k_{\mathrm{NI}}}{k_{\mathrm{IN}} + k_{\mathrm{IU}} + k_{\mathrm{NI}}} \tag{1}$$

where k_{NI} denotes transition rate from N to I, k_{IN} transition rate from I to N, and k_{IU} transition rate from I to U.

For transient hidden I state with high free energy, we assumed that $k_{\mathrm{NI}} \ll k_{\mathrm{IN}}$ and $k_{\mathrm{NI}} \ll k_{\mathrm{IU}}$, then Supplementary Equation (1) can be simplified as:

$$k_u = \frac{k_{\mathrm{IU}} k_{\mathrm{NI}}}{k_{\mathrm{IN}} + k_{\mathrm{IU}}} \tag{2}$$

All transition rates are force-dependent. We suppose that transition rates of k_{NI}, k_{IU}, and k_{IN} follow Bell's model:

$$k_{\mathrm{NI}}(f) = k_{\mathrm{NI}}^{0}\exp(fx_{\mathrm{NI}}/k_{B}T) \quad (3)$$

$$k_{\mathrm{IN}}(f) = k_{\mathrm{IN}}^{0}\exp(-fx_{\mathrm{IN}}/k_{\mathrm{B}}T) \quad (4)$$

$$k_{\mathrm{IU}}(f) = k_{\mathrm{IU}}^{0}\exp(fx_{\mathrm{IU}}/k_{\mathrm{B}}T) \quad (5)$$

where k_{NI}^{0} is zero-force transition rate from N to I, and x_{NI} denotes the transition distance from N to I, etc. Each transition rate has two parameters in Bell's model, and there are totally six parameters.

As shown in the Fig. 4a, Supplementary Equation (2) can fit the experimental unfolding rates over full force range with one set of parameters in Supplementary Table1. The fitting results k_{NI}^{0} = 0.046 s^{-1} and x_{IN} = 0.51 nm are consistent with Bell's model fitting result at forces ranging from 10 to 50 pN in Fig. 4a ($k_{u,1}^{0}$ = 0.032 s^{-1} and $x_{u,1}$ = 0.55 nm).

Meanwhile, the summation of transition distances x_{NI}, x_{IN} and x_{IU} gives the extension difference between N and TS2 states of 3.1 nm, which agrees with Bell's model fitting results at force range of 5-7 pN ($x_{u,2}$ = 3.1 nm, Fig. 4b).

Supplementary Table 1: One set of parameters in Supplementary Equations (2-5) to reproduce the force-dependent unfolding rate of Csp (Fig. 4a).

k_{NI}^{0} (s^{-1})	x_{NI} (nm)	k_{IN}^{0} (s^{-1})	x_{IN} (nm)	k_{IU}^{0} (s^{-1})	x_{IU} (nm)
0.046	0.51	38000	1.3	230	1.3

As the six parameters are not independent, different sets of parameters can fit the experimental results. Value range of dependent parameters are given in Supplementary Figure 8. From the viewpoint of free energy landscape (Fig. 4c), $k_{\mathrm{u}}(f)$ is not sensitive to the position and the free energy of I state, as long as the I state is located between the two transition states TS1 and TS2.

Supplementary Note 2

Estimation of the apparatus rate $\mathbf{k}_A$

The diffusion coefficient of paramagnetic bead $\mathrm{D} = \frac{\mathrm{k}_B T}{6\pi\eta\mathrm{r}}$, where η is the viscosity of solution, r is the radius of the Dynabeads M270. Then the value of D is about 1.6 x10^5 nm^2 s^{-1}. We calculated apparatus rate k_A with the equation[2]:

$$k_A = \frac{D(\kappa_F \kappa_\ddagger)^{\frac{1}{2}}}{2\pi \mathrm{k_B} T} \exp(-\Delta G^\ddagger/\mathrm{k_B}T) \quad (6)$$

where $\Delta G^\ddagger$ is the free energy barrier, κ_F and $\kappa_\ddagger$ denote the stiffness of the folded state and of the barrier, respectively.

Based on the folding/unfolding probability obtained in experiment (Fig. 2a and Supplementary Figure 10), we get $\kappa_F = 0.1\frac{\mathrm{k}_B T}{\mathrm{nm}^2}$, $\kappa_\ddagger = 0.08\frac{\mathrm{k}_B T}{\mathrm{nm}^2}$, and $\Delta G^\ddagger$= 1.3 $k_B T$. Therefore, the value of k_A is about 620 s^{-1}. The maximum unfolding rate in our force-jump experiments at 50 pN is 23 s^{-1} which is more than 20 times slower than k_A .

Supplementary Figures

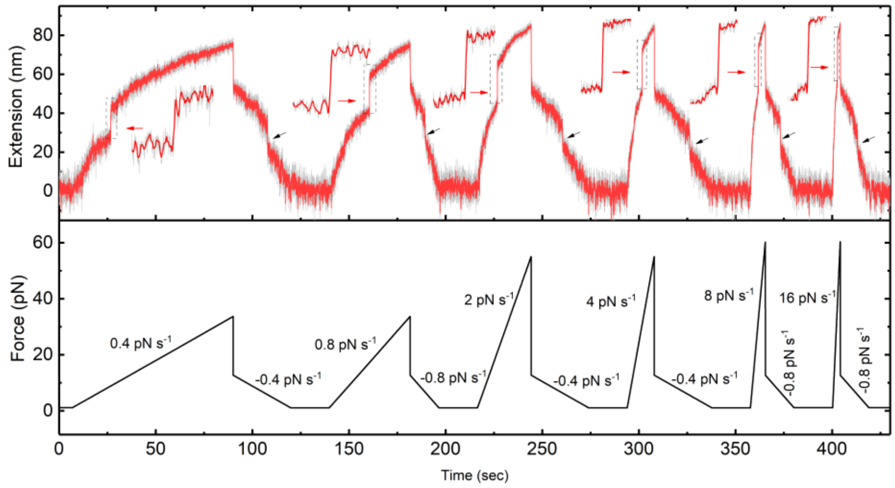

Supplementary Figure 1. Recorded extension time course when force is controlled with different loading rates**.** Unfolding and folding events of Csp can be detected by abrupt extension jumps. Zoomed in unfolding steps are shown in insets.

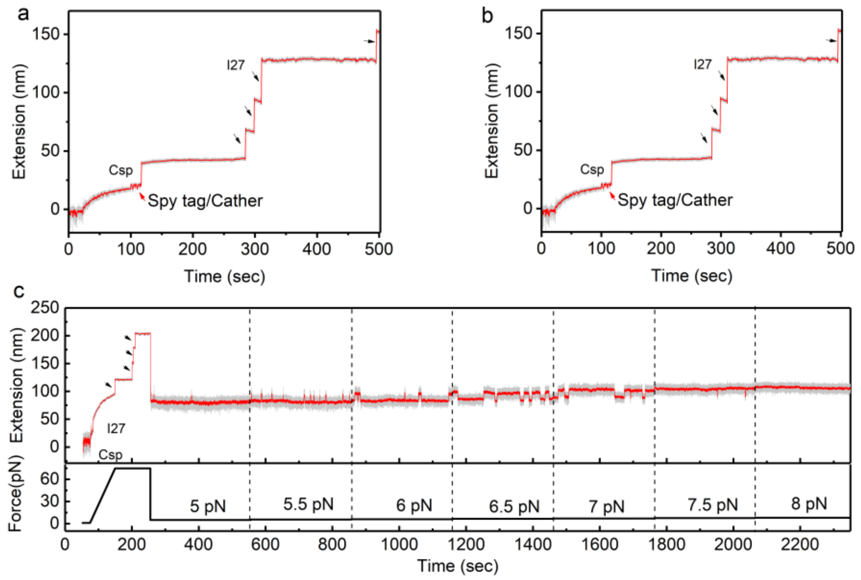

Supplementary Figure 2. Force-dependent unfolding of Csp at constant loading rates followed by constant-force measurement. **(a, b)** Unfolding of Csp was followed with four I27 unfolding steps. **(c)** Unfolding of Csp and four I27 domains was followed with equilibrium folding and unfolding dynamics of Csp at various constant forces from 5 pN to 8 pN.

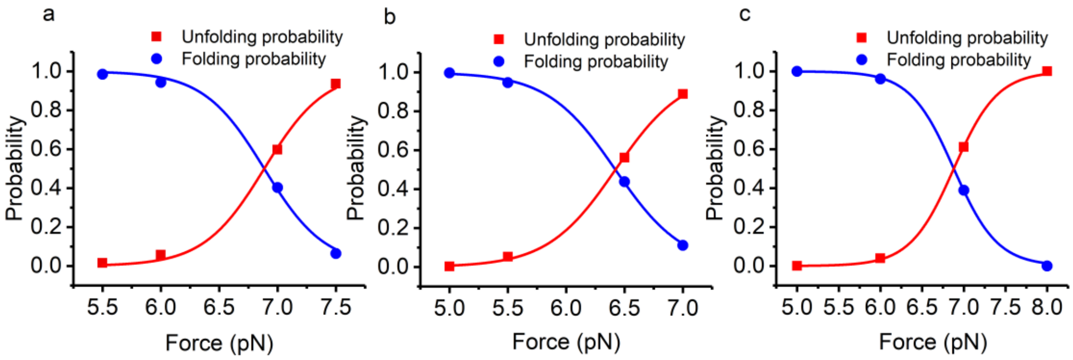

Supplementary Figure 3. Force-dependent folding and unfolding probability of Csp. **(a, b, c)** Three independent equilibrium measurements are shown.

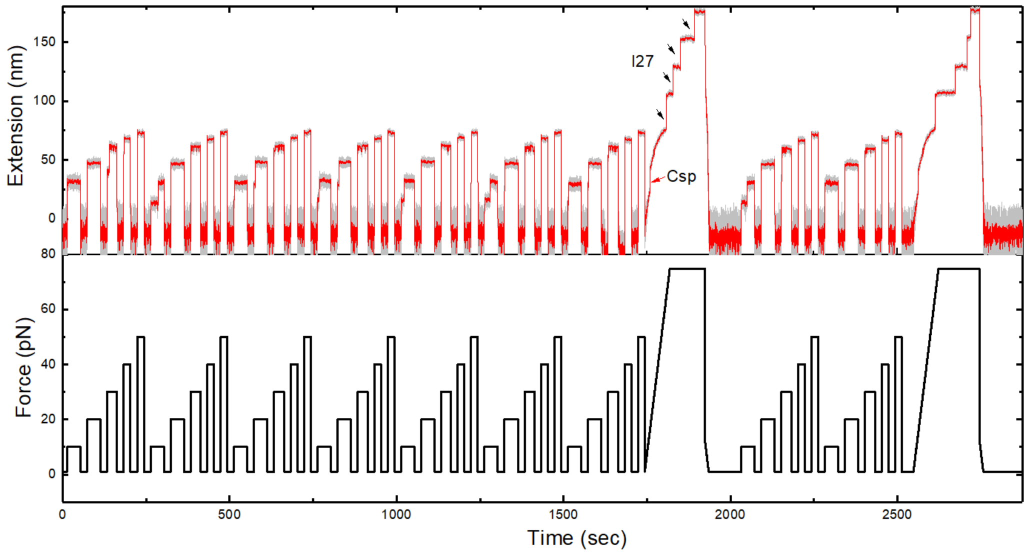

Supplementary Figure 4. Unfolding process of Csp at various constant forces recorded in force-jump experiment. After several cycles of measurements, force was increased to higher value to check the fingerprint unfolding signal of I27 domains.

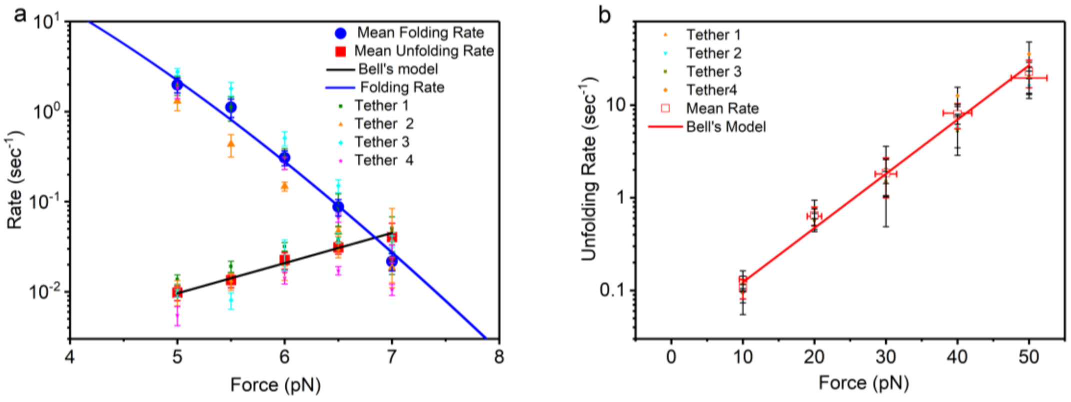

Supplementary Figure 5. Force-dependent folding rates and unfolding rates of Csp. (a) The average unfolding and folding rates of Csp measured by equilibrium constant force experiments from four different tethers. Totally 116/115, 79/80, 210/212, 219/221, 66/67 unfolding/folding events of Csp at forces from 5 pN to 7 pN were collected. **(b)** The average unfolding rates of Csp measured by force-jump experiment from four different tethers. Unfolding rates was calculated from 89, 96, 98, 99, 96 unfolding events for forces of 10 pN, 20 pN, 30 pN, 40 pN, and 50 pN, respectively. Error bar of rate shows the standard error of the mean, and force is estimated to have 5% uncertainty.

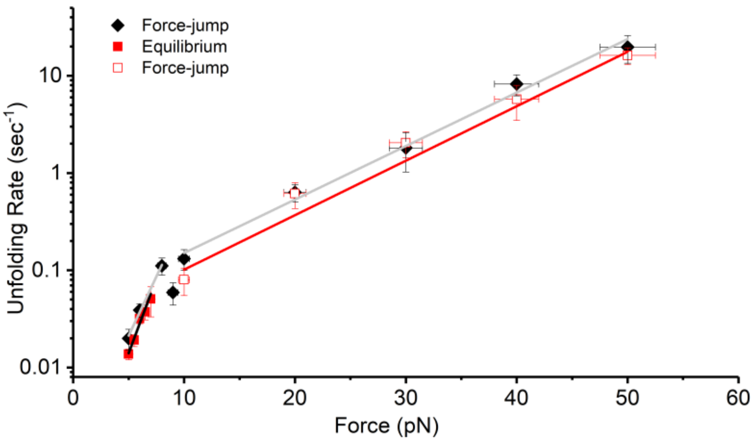

Supplementary Figure 6. Force-dependent unfolding rates obtained from force-jump measurement of one Csp tether in force range of 5-50 pN. The unfolding rates are on two linear lines with distinct slopes, which is consistent with the result from both equilibrium measurements and force-jump measurements in main text. Error bar of rate shows the standard error of the mean, and force is estimated to have 5% uncertainty.

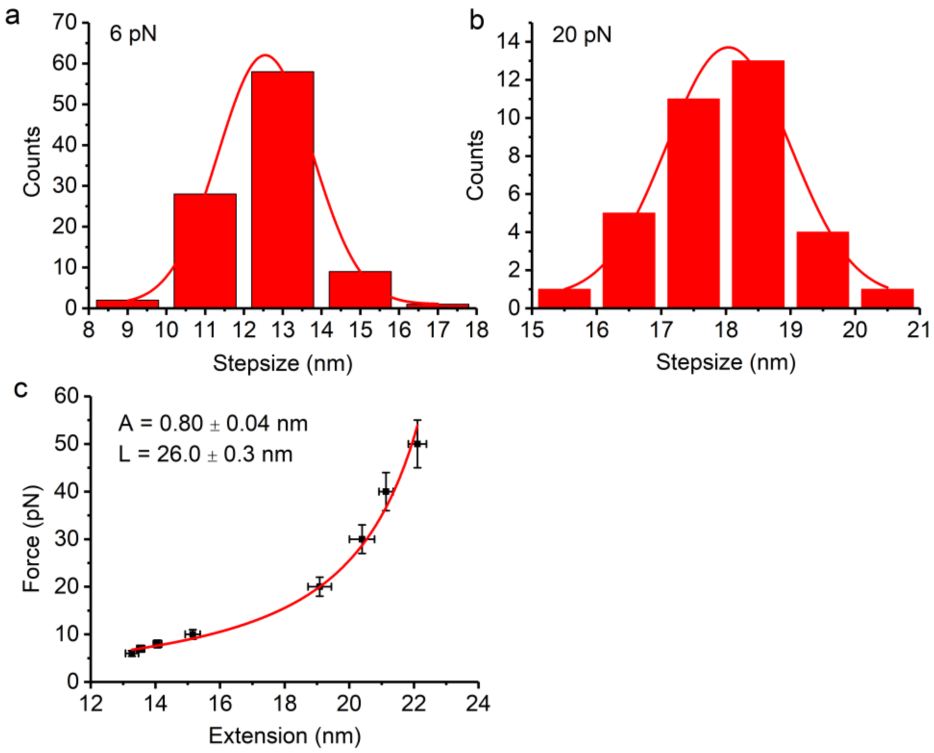

Supplementary Figure 7. Force-extension curve of unfolded peptide obtained from unfolding step size at different forces. **(a, b)** Histogram of unfolding step size at 6 and 20 pN for Csp, respectively. **(c)** Extension of unfolded peptide is obtained from extension of native state and unfolding step size. Worm-like chain fitting gives persistence length of 0.80 nm and contour length of 26.0 nm for unfolded polypeptide of Csp. Error bar of extension shows the standard error of the mean, and force is calculated to have 5% error.

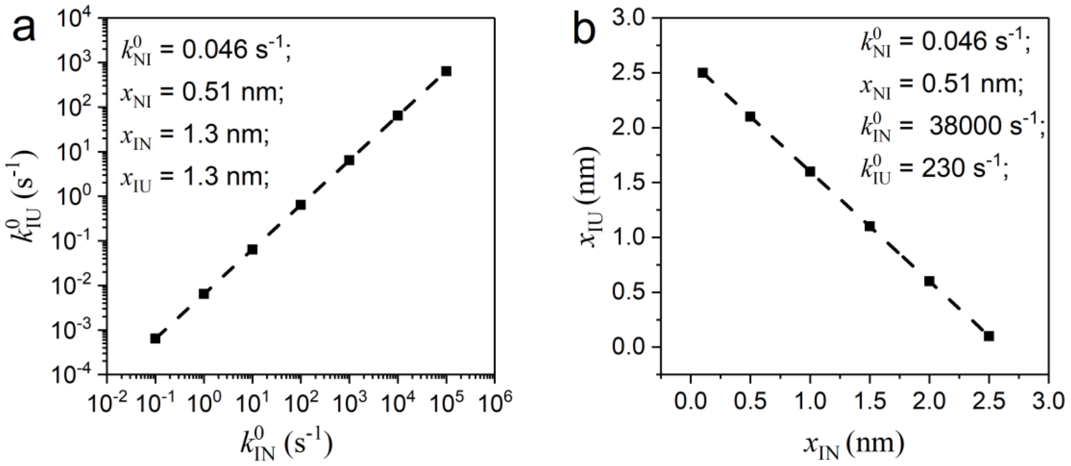

Supplementary Figure 8. The interdependence between parameters to fit force-dependent unfolding rate by NIU model. **(a)** The parameters were fixed to values in Supplementary Table 1, except for k^0_{IN} and k^0_{IU}, which were adjusted at the same time to fit force-dependent unfolding rates in Figure 4A. These two parameters show a linear relationship. **(b)** The same operation was applied to x_{IN} and x_{IU}. Linear relationship shows the summation of x_{IN} and x_{IU} is ~2.6 nm.

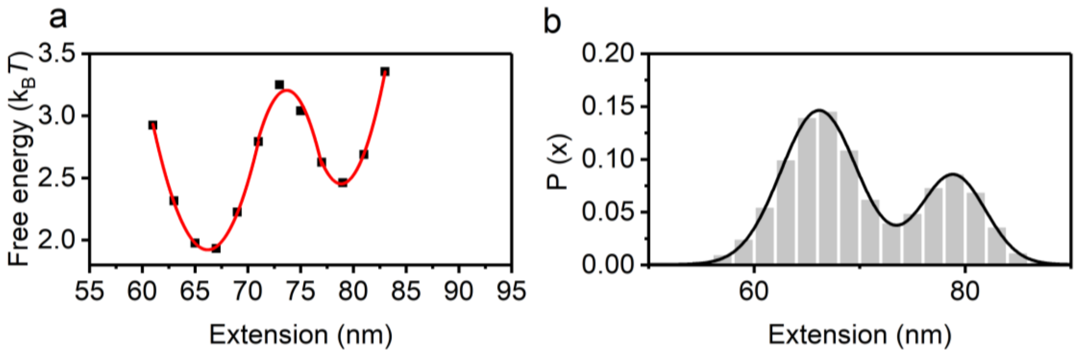

Supplementary Figure 9. Free energy as a function of extension **(a)** were obtained from the extension distribution (**b)** from equilibrium constant-force measurement at 6.3 pN. Quadratic fitting was done at the positions of free energy wells and barrier (red solid line).

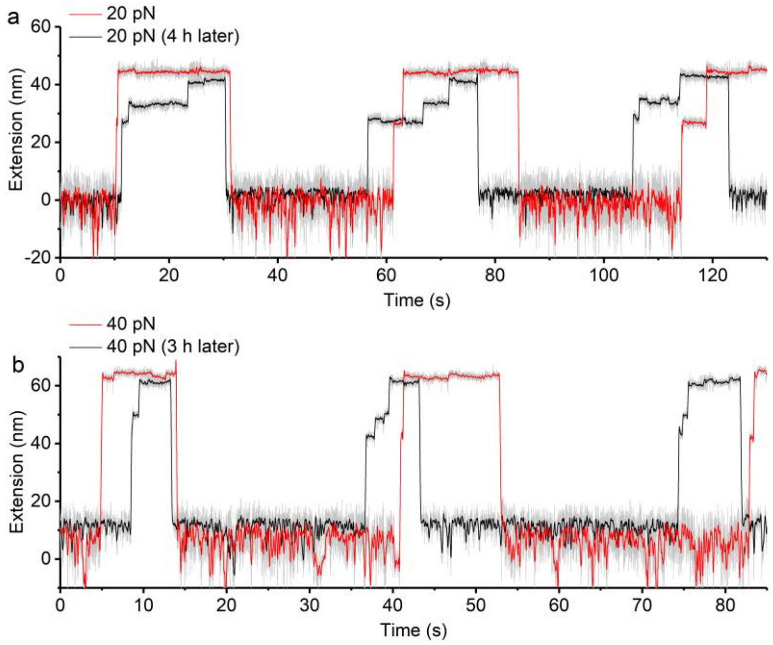

Supplementary Figure 10. Occasional observation of multi-step unfolding events of Csp after several hours measurement. **(a, b)** From the start of experiment, Csp unfolded with one step within three hours continuous measurement. Multi-step unfolding events of Csp started to be recorded in force-jump experiments after three hours.

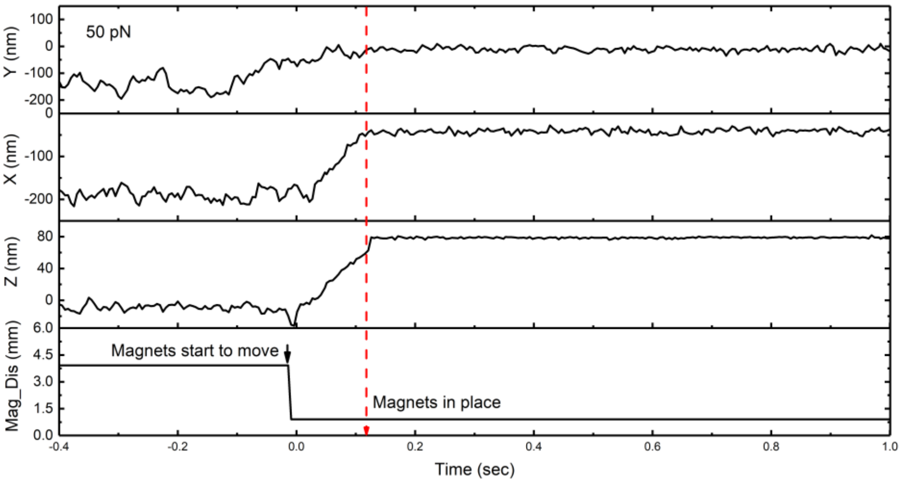

Supplementary Figure 11. Estimation of the time for magnets to arrive at the setting distance. The time course of the three-dimensional positions of the paramagnetic bead (X, Y, and Z) are recorded when magnets move closer to sample to increase the force to 50 pN (black arrow). It takes about 0.15 second to move to the setting position (red dashed line arrow).

1 Pierse, C. A. & Dudko, O. K. Distinguishing signatures of multipathway conformational transitions. *Phys. Rev. Lett.* **118**, 088101 (2017).

2 De Sancho, D., Schönfelder, J., Best, R. B., Perez-Jimenez, R. & Muñoz, V. Instrumental Effects in the Dynamics of an Ultrafast Folding Protein under Mechanical Force. *J. Phys. Chem. B* **122**, 11147-11154 (2018).
